# The science of printing and polishing 3D printed dentures

**DOI:** 10.12688/f1000research.157596.2

**Published:** 2025-01-06

**Authors:** Kavishma Sulaya, Swapna B V, Vaishnavi M Nayak

**Affiliations:** 1 Department of Prosthodontics and Crown & Bridge, Manipal College of Dental Sciences, Manipal Academy of Higher Education, Manipal, Karnataka, 576104, India

**Keywords:** Additive manufacturing, 3D printed dentures, Surface roughness, Chairside polishing, Glaze materials.

## Abstract

**Objective:**

To analyze the effectiveness of various techniques available for printing, finishing and polishing of 3D printed prosthesis.

**Methods:**

The articles were selected from electronic databases including PubMed and Scopus. Recently, lot of advancements have been observed in the field of 3D printing in dentistry.

**Results:**

Numerous studies were found explaining the factors affecting the surface roughness such as printing speed, direction, layer thickness, post curing, etc., and the significance in achieving a smooth surface finish of a 3D printed prosthesis. The methods employed to achieve this range, similar to conventional and chairside polishing, are to use advanced coating materials such as light cured glazes to nanoparticles.

**Conclusion:**

3D printing is being used in day-to-day practice and the prosthesis must be aesthetic looking to satisfy the patients’ expectations. There is a lack of data supporting any one polishing method for the prosthesis. There is a need for further research on the existing techniques and newer advancements yielding aesthetic prostheses with an optimal surface finish.

## Introduction

Three-dimensional (3D) printing has been introduced in the industrial sector since 1980’s. ^1^ Its advent in dentistry has led to the revolutionization of the field. The surface finish and polished surface of the prosthesis plays a vital role as there is an increased demand for esthetic restoration and life-like prostheses. This property is of clinical relevance not only for its esthetic reasons but also as it directly or indirectly aids in the retention of microbial plaque on its surface. Increased surface roughness results in high plaque accumulation, thereby increasing surface fatigue and reducing the biocompatibility of the material leading to oral candidiasis and denture stomatitis. Polishing of the prosthesis is either done by mechanical method (Conventional, chairside) or chemical method to reduce the adherence of the microorganisms. Coating materials can be used as an additional layer for additively manufactured prostheses as an alternative to conventional polishing.

The Surface roughness is determined using Ra or Rz values. Ra is the average value of the deviation of a measuring profile from a central line along the measuring length. ^2^ Rz is the deviation from the mean line focusing on the highest peak and valley. It is the average of the five individual values in sequence from individual roughness depth. Estimated Ra value can be calculated by dividing Rz value from 4-7 units, but estimated Rz is calculated by multiplying Ra value by 10-15. According to Fernandaz P et al, a surface roughness (Ra) of 0.2 μm was set as the clinically acceptable value. ^2^ The surface roughness is affected by the number of layers added, the speed of printing, the material used, the thickness of the material, the polishing technique, and operator’s skills. ^3^

Conventionally, laboratory polishing of the prosthesis is done with pumice slurry and buff followed using aluminum oxide polishing paste. Chairside polishing is done using various chairside kits such as the Shofu kit, JOTA polishing kit, microdont polishing kit, etc., from coarser to fine grit burs at the speed of 5000 rpm. The major disadvantage of these conventional methods is the dimensional change that may occur, increased risk of fracture etc. Chemical polishing includes placement of the denture base in an ultrasonic bath and coating it with another thin layer of resin, laser polishing, etc. ^4^

Additively manufactured prostheses are fabricated layer by layer which results in a lot of surface irregularities due to its stepwise effect that require adequate polishing. ^2^ A combination of multiple methods is required to finely polish the prosthesis to achieve the required results. There is no standard protocol available to polish a 3D printed prosthesis and the available literature to prove which method of polishing is superior to the other is scarce. Hence this narrative review focuses on understanding the 3D printing process and its effect on polished surface.

## Methods

The relevant articles were selected from two databases Pubmed and Scopus after conducting search using the keywords from 1995 to 2024. Recent reviews and original research articles which analysed the data on surface roughness, various polishing and 3d printing techniques were included.

## Discussion

### Additive manufacturing

Additive manufacturing (AM) is the method in which materials are combined layer by layer to create objects from 3D model data. ^5, 6^ The new successive layers are joined to the preceding layers by either melting, fusing, or polymerization processes. American Society for Testing and Materials classifies AM technology into seven categories based on the printing methods. ^7, 8^

1) VAT photopolymerization
2) Fused Deposition Modeling
3) Powder Bed Fusion
4) Material jetting or inkjet printing
5) Binder jetting or three-dimensional printing
6) Direct Energy Deposition
7) Manufacturing of laminated objects.

Some of the methods used for printing complete dentures include

*VAT photopolymerization*

*Stereolithography (SLA)*

This was developed in 1963 and was one of the first additive manufacturing method. This technique is based on light polymerization, in which a chain reaction between the resin and the monomer is initiated by an electron beam or UV light. The polymer resin used for printing dentures is supplied in liquid form which includes photopolymers. The building platform is submerged in a liquid polymer tank, which can be moved up, polymerizing the initial layer by laser beam. The building platform is lowered to descend into the tank to create a subsequent layer. This causes polymer liquid to cover the surface of the previously built layer, repeating the polymerization process as the platform moves. This process is repeated until all the layers are built and the 3D model is completed. The completed model is photo cured for increased strength. The models printed using SLA have high resolution and quality but are expensive and time consuming. ^9^ The thickness of each layer is controlled by two factors, i.e. energy of the light source and the amount of exposure. ^10, 11^

*Digital light processing (DLP)*

In additive manufacturing, digital light processing technology effectively cures photopolymer resin, especially in the dental industry. It projects a two-dimensional pixel pattern and cures entire resin layers at once employing a Digital Micromirror Device (DMD), which is made up of millions of mirrors that can independently switch between on and off states. As a result, build durations are decreased because they are solely influenced by exposure duration and layer thickness, not by layer size or the quantity of structures being constructed. Moving projectors, dual-projector configurations, and high-resolution DMD chips (e.g., 4K) are some of the methods used to preserve accuracy on bigger build platforms. Speed and precision are enhanced by innovations like Carbon3D's Continuous Direct Light Processing (CDLP) and Prodways' Moving Light technology. Systems that optimize object detachment throughout the build process, such as Force Feedback and VAT deflection feedback (VDFS), also improve productivity and print quality. ^11– 13^

The materials used and the process of printing are the same as SLA, but the source of light for polymerization used is different. Laser is used for polymerization in SLA, but a digital projector is used in DLP. DLP method prints at a faster speed compared to SLA, and intensity of the light source can be varied. ^11– 13^

*Fused depositing modeling (FDM)*

The polymer used in this method has thermoplastic properties, the layers can be joined together throughout the printing process and solidify at room temperature once the printing is complete. The most used materials are polylactic acid, acrylonitrile butadiene styrene, and polycarbonates. ^14, 15^ The material should have a low melting point, sufficient viscosity to flow out of the nozzle and adequate strength to support the next layers. High speed, easy processing, and low cost are the advantages of FDM. Limitations include low surface quality, layered appearance, and low mechanical strength. ^16^

*Material jetting*

It is an injection system in which the photopolymer goes through several nozzles to build the 3-dimensional model layer by layer. The material is cured by UV radiation and shares a chemical foundation with vat photopolymerization. ^17, 18^

### Clinical applications of different printing techniques in complete denture fabrication

SLA and DLP can be vicariously used for the fabrication in clinical and laboratory procedures such as custom tray fabrication, cast fabrication, trial dentures, definitive denture base and teeth. However FDM and MJ has limited applications in fabrication of custom trays and complete denture prototypes. ^19^

The accuracy and properties of the final product are affected by various printing and processing parameters and material composition. The printing process parameters include printing direction, layer thickness and post curing which affect the final printing product. ^10^

### Printing direction

The build direction/angle selection is vital, as it influences the amount of support the structures require which impacts the accuracy of the final product. The printing direction, which establishes the angle between the component layers and the applied force affects the mechanical qualities of the 3D printed denture. ^20^ Thus, selecting the right printing orientation is crucial for optimizing the mechanical properties of the final product. Yan S et al., evaluated the build angle (0°,45°, and 90°) on the surface characteristics of 3D printed complete denture base samples by DLP method and concluded that 0° built specimens exhibited significantly lower mean roughness when compared with 45° and 90°. They exhibited block-like protrusions on the surface which were evenly distributed and surrounded by depression. The highest mean surface roughness was seen in specimen built at 45° followed by 90° group, while the smoothest surface was seen with 0°. ^21^ Ataei et al., evaluated the surface roughness of 3D printed samples at 0° orientation and 100 μm layer thickness and later subjected them to thermocycling. They observed that polishing was an effective way to make layered structure disappear in 3D printed resin. There was no variation in the layered structure in the presence or absence of thermal stress. ^22^ Lee et al., evaluated the surface properties of 3D printed resins with DLP method in three distinct build angles (0,45°, and 90°) and found that the 0° build angle provided a smooth surface, regardless of the thickness. The highest surface roughness was observed at a 45° build angle. However, this study also indicated that decreasing thickness can enhance surface roughness. ^23^ Similarly, Shim et al., also concluded that 45° printing orientation showed the highest value of surface roughness compared with those of the other groups (0° and 90°). The surface obtained from the 0° printing orientation revealed craters of varied sizes, whereas the 45° orientation group displayed recurring oblique ridges. The 90° orientation group displayed asymmetrical surfaces that blended spherical and non-round geometries. ^24^

It is to be noted that studies on build orientation and layer thickness in additive manufacturing of denture materials often utilize standardized disc-shaped specimens. When these specimens are oriented horizontally (0°) and sliced, certain surfaces, including those tested, may not exhibit the stepwise effect characteristic of additive manufacturing. ^25^ This limitation arises because denture morphology features irregular geometries. It is not conclusive that horizontally printed dentures inherently have superior surface properties. Additionally, in horizontal orientations, the influence of layer thickness on surface morphology is primarily observed on the sidewalls rather than the tested surfaces.

### Layer thickness

The layer thickness (LT) is the measurement of the thickness of each slice of the part that builds upon the layer before it. The layer thickness affects the accuracy and other properties of the final denture. ^26^ Lee et al., evaluated the effect of two different layer thickness (50 and 100 μm) on the surface roughness of 3D printed resins with DLP method. They observed that the surface smoothness achieved with a 100μm layer thickness and 0° built angle was similar to 50 μm layer thickness. These settings could facilitate effective fabrication with favorable surface smoothness, as products with a 100 μm LT could be fabricated faster. ^23^

### Post curing

3D printed resins are photo-polymerized material, and post-curing time affects the performance of the material. The photo initiator initiates the polymerization, using the laser or light projector resulting in partial curing. The unbonded monomers then join to form a polymer, which further develops into a cross-linked macromolecule. The primary surface texture is now developed. The polymerization process is completed by further curing using a light cure unit as a post curing step. The post-curing period as recommended by the manufacturers varies from 20 to 60 minutes to ensure complete polymerization of the material. ^27, 28^ Ahmed et al., concluded that 30 minutes is the minimal post-curing time to obtain optimal results since additional curing had no discernible impact on the material’s characteristics. ^29^ Ping li et al., studied the surface topography and roughness of 3D printed resins after various post-curing methods using different devices and observed no significant differences between the post-cured specimens with various devices. Significantly lower Ra values were exhibited by the 3D printed specimens than the conventional poly methyl methacrylate (PMMA). ^30^

### Polishing techniques

**Figure 1.**
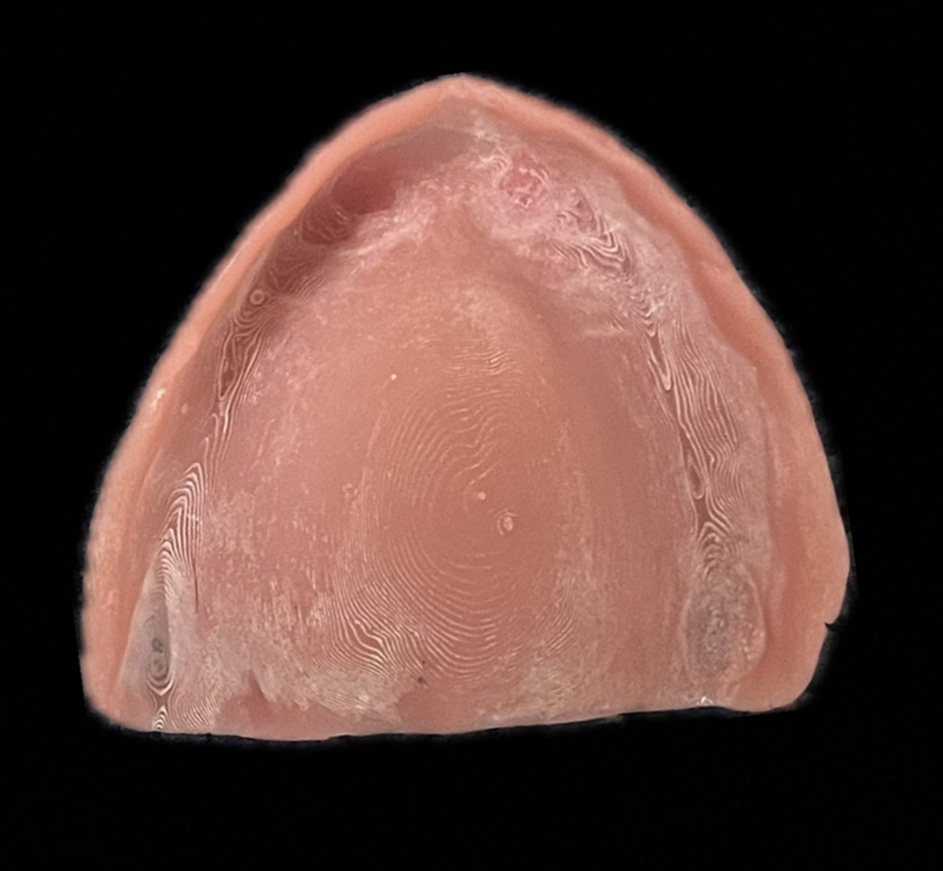
Stepwise effect in 3D printing.

In day-to-day clinical practice, acrylic dentures are subjected to modifications by trimming which would create a rougher surface. ^4, 31, 32^ To smooth out rough layers, polishing these acrylic dentures is recommended to reduce the free surface energy. The polished surface improves hygiene of denture as it prevents microbial adherence and colonization. ^4, 33^ Routinely, in a laboratory setting acrylic dentures are polished with pumice slurry followed by aluminum oxide polishing paste (Al _2_O _3_). ^4, 33, 34^ With the advent of digital dentistry, 3D printed dentures are being routinely used for fabrication of removable dentures. Inherently, 3D printed dentures have layers due to their printing nature affecting the surface properties. The increase in the surface roughness of 3D printed dentures could be attributed to the edge stepwise effects. ( Figure 1) Additionally, the chairside corrections done during clinical procedures, adds on to the surface roughness. Although the literature on the effect of conventional laboratory polishing techniques on surface roughness of 3D printed dentures is limited, the currently available studies have shown clinically acceptable smooth surfaces which prevents sequelae of plaque accumulation.

Fernandez et al., compared the surface characteristics of 3D printed and milled denture base materials with conventional heat-cure resins. ^2^ The study compared resins that were polished or coated to different degrees, such as the *intermediate polishing group* (pre-polishing with grinding paper 150,180 grit size; brushed along with pumice slurry followed by polishing paste using lathe bristle brush), *high gloss polish group* (procedure similar to intermediate group, additionally the resin block was polished with polishing paste and soft cloth wheel to attain high gloss), coated resin group (thinly coated with unpolymerized resin for 3D printing procedure and cured with a light curing unit), and coated and polishing (combined procedure of coated and high gloss polished group). ^2^ They concluded that polishing (high gloss), coating and coating along with polishing resins were within the clinically relevant surface roughness threshold. ^2^ The high gloss polished 3D printed resins provided lower roughness compared to coated specimens with clinically relevant lower Ra values. ^2^ The polishing of 3D printed dentures showed superior Ra values when compared to milled dentures.

Routinely chairside manipulation of the denture is required leading to practitioners using chairside polishing kits for polishing. Chairside polishing kit has been used as a substitute method of choice in situations where laboratory polishing cannot be performed. ^35^ With conventionally used heat cured PMMA, the chairside polishing kits have provided clinically acceptable results although inferior to laboratory polishing. ^35^

Al-Dulaijan et al., compared laboratory and chairside polishing on 3D printed denture resins and concluded that laboratory polishing showed lower Ra values than chairside and unpolished denture base resins. ^4^ Sandpaper of grit size 1000 and 2500 were used for finishing procedures. The specimens for laboratory polishing group were then polished with pumice slurry and brush for 90 seconds at 1500 rpm, followed by universal polishing paste and lathe brush for 15 seconds at 3000 rpm respectively. For chairside polishing three commercially available kits Microdont, Acrypoint and Shofu were used with 3 polishing burs of coarse, medium and fine grain for a minute each. Although conventional polishing showed superior results, the author concluded that chairside polishing kits are an efficient technique when access to laboratory method is limited. ^4^ Quezada et al., investigated the surface roughness of Computer aided designing and Computer aided manufacturing (CAD/CAM) denture resins to conventional PMMA using chairside polishing kit JOTA and found that 3D printed resin specimen showed lower Ra values to conventional heat cured dentures. ^3^

### Coating materials on 3D-printed resins

To decrease surface roughness, light-cured glazes and coatings have been applied to digitally manufactured dentures. ^36– 38^ Additionally, it reduces surface porosity and imperfections, prevents microleakage, and strengthens resin and increases resistance to stains. ^36– 38^ Dickinson et al., reported that a low viscosity sealant lowered the surface roughness in acrylic resins (Ra values = 0.2 μm). ^3, 39^ Pigmented light cure glaze materials can also be used for the characterization of teeth and gingiva masking the monolithic appearance of the dentures. Silica and titanium nanoparticles are incorporated in dental glaze materials which improve the resin’s surface properties and lower the surface energy, reducing biofilm adhesion. ^36, 40^ Choi et al., evaluated the surface of different light polymerized denture glaze materials and concluded that the glaze materials lowered the surface roughness values. ^36^ Ra values varied from 0.26 to 0.15 μm exceeding the threshold limit. ^36^ Along the acceptable polished surface, glaze materials with nanoparticles such as silica dioxide and titanium dioxide also improved the surface hardness and elastic modulus. ^36, 41^ This could be attributed to coupling agents such as 3-trimethoxysilylpropyl methacrylate which creates a bond between both the inorganic and organic phases of acrylic resins. ^36^

**Figure 2.**
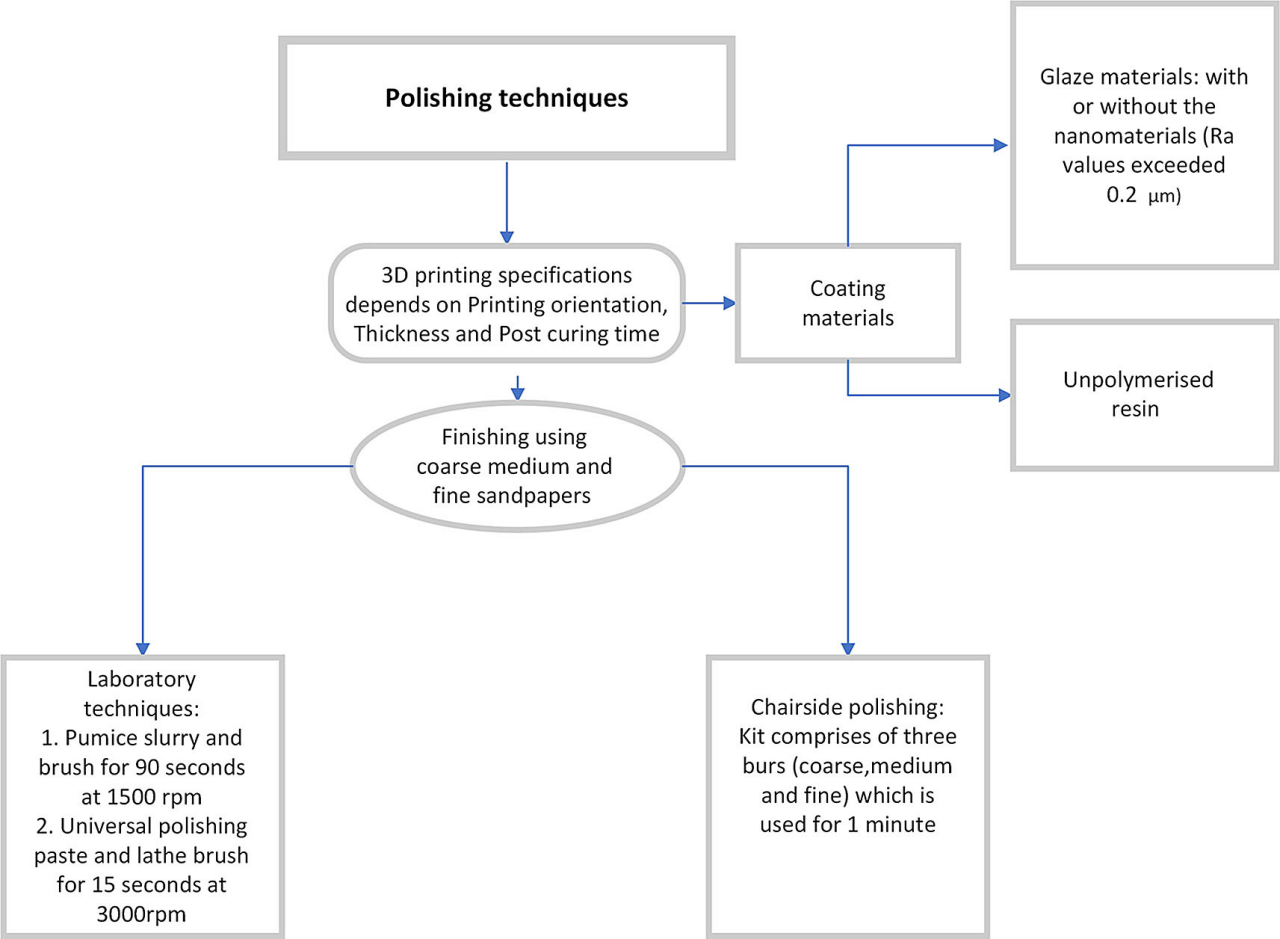
Schematic representation of various methods of polishing 3D printing dentures.

Fernandez et al., evaluated surface roughness of additively fabricated dentures coated with the same unpolymerized material. ^2^ Coated specimens demonstrated Ra values of 0.16 μm well within the clinically relevant threshold of 0.2 μm. Although, solely coated specimens did not yield any superior outcome to solely polished specimens. ^2^ The coated and polished specimens gave superior results. ^2^ However, coating with unpolymerized material to 3D printed dentures may be regarded as an alternative to conventional polishing. ^2^ Various polishing methods have been summarised in Figure 2.

## Conclusions

Surface roughness of the printed prosthesis depends on the printing orientation, thickness of the layer and the post-curing process. Advised post-curing time is 20 to 60 minutes, following which polishing can be done by conventional laboratory or chairside methods. Light cured glaze coating materials with incorporated nanomaterials provide acceptable surface roughness values along with better surface hardness and modulus of elasticity. However, the current studies conclude that conventional laboratory polishing of 3D printed dentures is sufficient for achieving a clinically acceptable polish. Still, there is a need for a standardized protocol for finishing and polishing 3D printed prostheses and the potential for various coating materials to be used as an alternative to the conventional polishing method.

## Data Availability

No data are associated with this article.
