# Supplementary material for: The science of printing and polishing 3D printed dentures
Source: F1000Res. 2025 Jan 6;13:1266. Originally published 2024 Oct 23. [Version 2] doi: 10.12688/f1000research.157596.2 (PMC11748432; doi:10.12688/f1000research.157596.2)
Supplement: Peer review history

# Reviewer report: Reviewer response for version 2

**DOI:** 10.5256/f1000research.176341.r356575

**Published:** 2025-01-17

**Authors:** Shizhu Bai

**Affiliations:** 1 Air Force Military Medical University, Shaanxi, China

There is no additional comments.

Is the review written in accessible language?

Yes

Are all factual statements correct and adequately supported by citations?

Partly

Are the conclusions drawn appropriate in the context of the current research literature?

Yes

Is the topic of the review discussed comprehensively in the context of the current literature?

Yes

Reviewer Expertise:

Digital dentistry; 3D printing

I confirm that I have read this submission and believe that I have an appropriate level of expertise to confirm that it is of an acceptable scientific standard.

# Reviewer report: Reviewer response for version 1

**DOI:** 10.5256/f1000research.173059.r342684

**Published:** 2024-12-16

**Authors:** Shizhu Bai

**Affiliations:** 1 Air Force Military Medical University, Shaanxi, China

This article presents a well-structured review on 3D-printed denture techniques, analyzing printing and polishing methods and their impact on surface roughness, though some details could be further elaborated.

 'Some of the methods used for printing complete dentures include…'

 Can these technologies, such as FDM and MJ, be used directly to fabricate dentures for clinical use or only for making trial dentures or custom trays? It is recommended that this part be combined with practical clinical applications.

 DLP is the most commonly used printing technology and should be introduced in more detail.

 The author states that '... which impacts the precision of the final product'. According to ISO 5725-4, accuracy includes both trueness and precision. Does the author mean accuracy? 

 Currently, studies on build orientation and layer thicknesses are carried out using standardized specimens made of denture material in the form of discs. When such specimens are placed and sliced horizontally (0°), the stepwise effect is not generated on some of their surfaces, possibly including the surface being tested. However, the morphology of dentures is an irregular geometry, and the authors are advised to specify this, and it cannot be argued that the surface properties of horizontally printed dentures are more advantageous.

 Similarly, when the specimen is placed horizontally, the effect of the layer thickness on the surface morphology will not be demonstrated on the surface tested but on the sidewalls.

Is the review written in accessible language?

Yes

Are all factual statements correct and adequately supported by citations?

Partly

Are the conclusions drawn appropriate in the context of the current research literature?

Yes

Is the topic of the review discussed comprehensively in the context of the current literature?

Yes

Reviewer Expertise:

Digital dentistry; 3D printing

I confirm that I have read this submission and believe that I have an appropriate level of expertise to confirm that it is of an acceptable scientific standard, however I have significant reservations, as outlined above.

# Response

**Published:** 2024-12-24

**Authors:** Vaishnavi M Nayak

**Affiliations:** Department of Prosthodontics, Manipal Academy of Higher Education, Manipal, Karnataka, India

Thank you for your insightful feedback and constructive comments on our manuscript. We appreciate the opportunity to improve our work and have made significant revisions based on your suggestions. In response, we have made the following adjustments:

**Overall**

Comment from reviewer :

1. 'Some of the methods used for printing complete dentures include…'
  Can these technologies, such as FDM and MJ, be used directly to fabricate dentures for clinical use or only for making trial dentures or custom trays? It is recommended that this part be combined with practical clinical applications.
2. DLP is the most commonly used printing technology and should be introduced in more detail.
3. The author states that '... which impacts the precision of the final product'. According to ISO 5725-4 , accuracy includes both trueness and precision. Does the author mean accuracy? 
4. Currently, studies on build orientation and layer thicknesses are carried out using standardized specimens made of denture material in the form of discs. When such specimens are placed and sliced horizontally (0°), the stepwise effect is not generated on some of their surfaces, possibly including the surface being tested. However, the morphology of dentures is an irregular geometry, and the authors are advised to specify this, and it cannot be argued that the surface properties of horizontally printed dentures are more advantageous.
  Similarly, when the specimen is placed horizontally, the effect of the layer thickness on the surface morphology will not be demonstrated on the surface tested but on the sidewalls.

Response:

 Thank you for the thorough feedback, the following additions have been made:

1. We have added the clinical applications of different 3D printing techniques.
2. The DLP technique is elaborated in detail.
3. The term is corrected to accuracy.
4. The studies on build orientation and variation in morphology of dentures to that of the sample geometry are specified.

# Reviewer report: Reviewer response for version 1

**DOI:** 10.5256/f1000research.173059.r334672

**Published:** 2024-10-28

**Authors:** Vidya Shenoy

**Affiliations:** 1 Department of Prosthodontics, AJ Institute of Dental Sciences, Mangalore, India

Article is satisfactory and currently, the manuscript presents a well-structured and insightful analysis, contributing meaningfully to the field. Article covers relevant methods of polishing 3D  dentures.  There is a comprehensive discussion of all the methods including glazing. The facts are adequately supported by the current literature. Overall, it meets approval standards. Recommended for indexing

Is the review written in accessible language?

Yes

Are all factual statements correct and adequately supported by citations?

Yes

Are the conclusions drawn appropriate in the context of the current research literature?

Yes

Is the topic of the review discussed comprehensively in the context of the current literature?

Yes

Reviewer Expertise:

GERIATRIC DENTISTRY

I confirm that I have read this submission and believe that I have an appropriate level of expertise to confirm that it is of an acceptable scientific standard.
